# Epigenetic determinants of space radiation-induced cognitive dysfunction

**DOI:** 10.1038/srep42885

**Published:** 2017-02-21

**Authors:** Munjal M. Acharya, Al Anoud D. Baddour, Takumi Kawashita, Barrett D. Allen, Amber R. Syage, Thuan H. Nguyen, Nicole Yoon, Erich Giedzinski, Liping Yu, Vipan K. Parihar, Janet E. Baulch

**Affiliations:** 1 University of California Irvine, CA 92697, USA

## Abstract

Among the dangers to astronauts engaging in deep space missions such as a Mars expedition is exposure to radiations that put them at risk for severe cognitive dysfunction. These radiation-induced cognitive impairments are accompanied by functional and structural changes including oxidative stress, neuroinflammation, and degradation of neuronal architecture. The molecular mechanisms that dictate CNS function are multifaceted and it is unclear how irradiation induces persistent alterations in the brain. Among those determinants of cognitive function are neuroepigenetic mechanisms that translate radiation responses into altered gene expression and cellular phenotype. In this study, we have demonstrated a correlation between epigenetic aberrations and adverse effects of space relevant irradiation on cognition. In cognitively impaired irradiated mice we observed increased 5-methylcytosine and 5-hydroxymethylcytosine levels in the hippocampus that coincided with increased levels of the DNA methylating enzymes DNMT3a, TET1 and TET3. By inhibiting methylation using 5-iodotubercidin, we demonstrated amelioration of the epigenetic effects of irradiation. In addition to protecting against those molecular effects of irradiation, 5-iodotubercidin restored behavioral performance to that of unirradiated animals. The findings of this study establish the possibility that neuroepigenetic mechanisms significantly contribute to the functional and structural changes that affect the irradiated brain and cognition.

NASA and other space agencies are currently developing the technologies necessary to allow manned missions to a near-Earth asteroid, the Moon and then to Mars by the 2030’s. Among the obstacles to overcome in achieving these goals is protecting astronauts from the dangers of the deep space environment. One of those dangers is exposure to space radiation, which is comprised of protons and high (H) atomic number (Z) and energy (E), or HZE particles, a spectrum of which define galactic cosmic radiation (GCR). In particular, HZE particles are densely ionizing and highly damaging to cells and tissues^1^. These effects are exacerbated by the secondary ionizations known as delta rays that can extend millimeters from the primary particle track^1^. Space relevant fluences of these radiations, typical of those to which an astronaut would be exposed on a deep space mission to Mars, have been shown to induce severe cognitive impairments^2^,^3^,^4^. Coincident with radiation-induced cognitive dysfunction are other functional and structural changes in the brain, including elevated oxidative stress, neuroinflammation, and degradation of neuronal structure and synaptic integrity^2^,^5^. However, the molecular mechanisms by which space radiation exposures induce these dramatic alterations in CNS function remain relatively unexplored.

Proper function of the brain is multifactorial and among those parameters critical to cognition are epigenetic mechanisms, particularly DNA methylation and histone modifications. In recent years, the field of neuroepigenetics has seen significant advancements^6^,^7^ and compelling evidence suggests that persistent changes in DNA methylation may significantly impact learning and memory^8^,^9^,^10^,^11^,^12^,^13^. Specifically, rats treated with 5-azadeoxycytidine (5-aza) or zebularine (zeb) to inhibit DNA methyltransferase (DNMT) enzyme activity, or with the direct DNMT inhibitor RG108, failed to display normal memory stability^8^, reward learning^9^, or spatial learning and memory^10^,^11^. Using a different approach to manipulate DNA methylation, another study provided evidence that animals maintained on a methyl donor deficient diet exhibited altered expression of glutamate receptor related genes, as well as impaired novel object recognition and fear extinction^13^. Given issues that have been raised regarding the lack of chemical stability and toxicity, as well as a lack of selectivity of DNMT inhibitory drugs for specific methylating enzymes^10^,^14^,^15^,^16^, genetic manipulation of particular DNA methylating enzymes has provided a new and valuable strategy for evaluating the causal relationship between DNA methylation and learning and memory. A series of studies have demonstrated through the use of viral-mediated, localized gene knockdown that several writers and erasers of DNA methylation, including DNMT3a, and ten-eleven translocation methylcytosine dioxygenase (TET) enzymes TET1 and TET3 are involved in memory formation and addiction behavior^6^,^17^,^18^.

A variety of DNA modifications have been identified, but the most widely recognized and studied is 5-methylcytosine (5mC) whose conversion from cytosine is mediated by the activity of DNMT enzymes. The accumulation of 5mC at specific gene promoters has generally been associated with transcriptional repression; however, emerging evidence indicates that patterns of 5mC are dynamic and can also accumulate in distal regulatory elements and in gene bodies, leading to increased gene expression. DNA methylation can self-perpetuate though the activity of maintenance DNMT (DNMT1), which is important for proliferating cells. In the case of terminally differentiated neurons in the adult brain DNMT3a and 3b are particularly important, as *de novo* methyltransferase activity has been shown to modify previously unmarked cytosines in the genome. Specifically DNMT3a levels remain high in post-mitotic neurons, implying an important function in the adult brain^6^,^7^,^14^. 5mC can be passively oxidized or actively modified by TET enzymes. Among the TET family of proteins, TET3 is found to be most prevalent in the CNS and has been linked to learning and memory function^6^. Similarly, the potential significance of TET1 has been associated with altered DNA methylation patterns in response to neuronal activity^17^. While less is known about TET2, it is generally thought to be involved in developmental processes^19^. The primary oxidation product of 5mC is 5-hydroxymethylcytosine (5hmC), a stable modification that is found in the brain at higher levels than any other organ of the body^6^,^7^. Despite its stability in the brain, 5hmC can also be actively deaminated, with subsequent the recruitment of DNA repair mechanisms required to return the base to an unmarked cytosine.

5hmC modifications are enriched in intragenic regions of brain-specific and neuronal differentiation-associated genes where they correlate with gene expression, not silencing^19^. In concordance with the finding that 5hmC is an active epigenetic mark in neuronal tissue where it is found predominantly in euchromatin^20^, the dynamic accumulation of 5hmC has been shown to play an important role in learning and memory^17^,^18^,^21^. Therefore, these reversible epigenetic regulators might represent a dynamic mechanism for cellular plasticity in the response to external stimuli^22^,^23^, where radiation exposure may confound cognitive function during deep space exploration^24^. In fact, a recent study demonstrated that low dose space radiation exposure affected persistent changes in 5hmC levels that were associated with significantly altered expression of genes involved in neurodegenerative diseases and with cognitive impairment^25^.

Not only has inhibition of DNA methylation been shown to be beneficial in the treatment of cancer^26^, data also suggest that it may be an effective neuroprotection strategy, reversing DNA methylation patterns associated with the molecular and clinical manifestations of a variety of neuropathologies including anxiety, depression, Huntington’s disease and epilepsy^27^,^28^,^29^,^30^,^31^. Taken together, these findings provide the basis for the hypothesis that neuroepigenetic mechanisms may underlie, at least in part, radiation-induced cognitive deficits. In this study, we inhibited DNA methylation by disrupting S-adenosylmethionine (SAM) metabolism to test the hypothesis that exposure to a space relevant dose of ^28^Si particles causes changes in DNA methylation in the brain that drive radiation induced impairments in learning and memory.

## Results

### Experimental design

**Figure 1:**
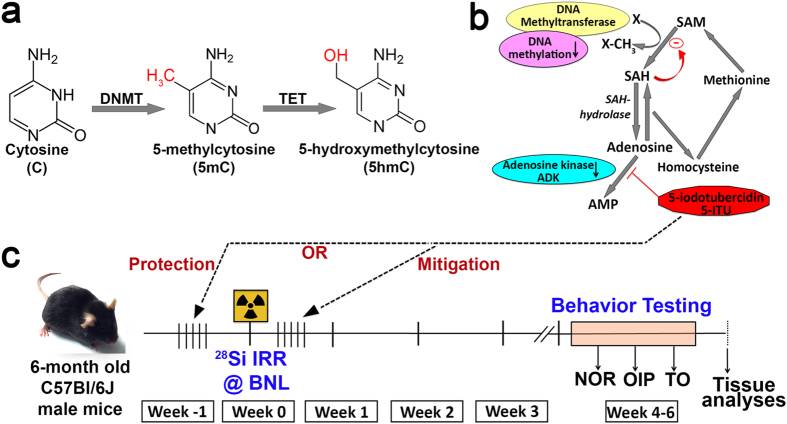
Experimental Design. (**a**) DNA methyltransferases (DNMT) convert cytosine to 5-methylcytosine (5mC) and ten-eleven translocation (TET) enzymes convert 5mC to 5-hydroxymethylcytosine (5hmC). (**b**) DNA methylation is linked to the S-adenosylmethionine (SAM) dependent transmethylation pathway that is regulated by adenosine under the control of adenosine kinase (ADK). DNMT uses SAM as a methyl donor, reducing it to S-adenosylhomocysteine (SAH) and then to adenosine and homocysteine (HCY) by SAH hydrolase. This process is dependent on removal of adenosine and HCY. Blockage of this pathway by 5-Iodotubercidin (5-ITU) and buildup of adenosine inhibits DNMT activity and reduces DNA methylation. (**c**) Schematic of the experimental time line. Mice in the protection study received 5-ITU treatments on 6 consecutive days pre-irradiation with the last injection 30 minutes prior to irradiation (^28^Si particles, 600 MeV/n, 20cGy at the Brookhaven National Laboratory, BNL). Mice in the mitigation study received 5-ITU treatments on 6 consecutive days post-irradiation with the first injection 30 minutes after irradiation. One month post-irradiation mice were administered behavioral testing (weeks 4–6) on the novel object recognition (NOR), object in place (OiP) and temporal order tasks (TO), after which brains were harvested for tissue analyses.

DNA methylation status depends on enzymatic reactions by DNA methyltransferases (DNMTs) that add methyl groups to cytosine bases in DNA to form 5mC and on the ten-eleven-translocation (TET) enzymes that perform oxidative reactions to convert 5mC to 5hmC (Fig. 1a)^32^. DNA methylation is inherently linked to the s-adenylmethionine (SAM) -dependent transmethylation pathway that is regulated by adenosine and glycine and under the control of adenosine kinase (ADK; Fig. 1b). DNMT uses SAM as a methyl donor, reducing it to S-adenosylhomocysteine (SAH) and then to adenosine and homocysteine (HCY) by SAH hydrolase. This process is dependent on continuous removal of adenosine and HCY. Blockage of this pathway and buildup of adenosine inhibits DNMT activity and subsequently reduces DNA methylation. 5-Iodotubercidin (5-ITU) is a potent inhibitor of ADK and its use results in the inability to clear adenosine^28^,^33^. Consequently, 5-ITU can be utilized as an effective inhibitor of methylation reactions including DNA methylation^32^,^33^. For the radiation protection study, mice were injected with 5-ITU for 5 days prior to irradiation (Fig. 1c). On the day of irradiation the mice received a final drug treatment 30 minutes prior to exposure. In order to similarly test the ability of 5-ITU to mitigate the effects of irradiation the mice received their first dose of 5-ITU 30 minutes after irradiation and for 5 subsequent post-irradiation days.

### Cognitive Testing

Beginning four weeks post ^28^Si particle irradiation (20cGy) all exposed and concurrent sham irradiated mice were subjected to a panel of three behavioral tasks. The novel object recognition (NOR) and the object in place (OiP) tasks depend on both the hippocampus and perirhinal cortex and test the animal’s ability to discriminate novelty (object or place)^34^,^35^,^36^. Additionally, we used the temporal order (TO) task to test an animal’s ability to differentiate between two familiar objects presented at different time intervals. The TO task depends on intact function of the perirhinal cortex and tests recency memory where an animal shows preference for the object presented less recently rather than the object more recently explored^34^,^35^. Data from each of these behavior tasks are presented as a discrimination index (DI) that is calculated as ([Novel location exploration time/Total exploration time] – [Familiar location exploration time/Total exploration time]) × 100. The DI provides a measure of an animal’s ability to discriminate novel versus familiar (objects or spatial locations). Animals with intact brain function spend more time exploring novel objects or object placements as compared to familiar objects or placements. A positive DI reflects this preference for novelty. Cognitively impaired animals have been shown to exhibit a lack of preference for novelty or even a preference for the familiar that is reflected by a low or negative DI^34^,^35^,^36^. The novel and familiar exploration times for each individual test phase as well as two-way ANOVA data are provided in the Supplementary Tables.

**Figure 2:**
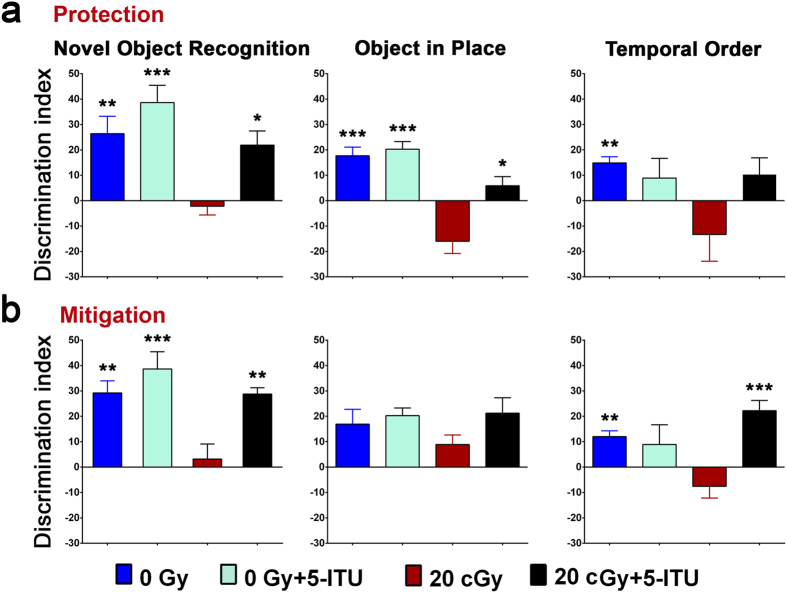
Protection and mitigation of radiation induced cognitive dysfunction by 5-ITU. (**a**) During the protection study, irradiation using 20cGy of ^28^Si particles impaired exploration on the novel object recognition (NOR), object in place (OiP) and temporal order (TO) tasks 1 month post exposure, but 5-ITU treatment protected against these adverse effects in the NOR and OiP tasks (20cGy + 5-ITU). (**b**) During the mitigation study, irradiation using 20cGy of ^28^Si particles impaired exploration on the NOR and TO tasks, but 5-ITU mitigated these adverse effects. All data are presented as mean ± SEM (N = 8–10 animals per group). *P* values are derived from ANOVA and Bonferroni’s multiple comparisons test. *P < 0.05, **P < 0.01, ***P < 0.001 for 0Gy, 0Gy + 5-ITU and 20cGy + 5-ITU as compared to 20cGy.

Prior to the NOR behavioral task, each mouse was habituated in the arena without stimuli. For this task, the total exploration time during the familiarization phase for each object was not different between any of the four experimental groups. Five minutes later, in the NOR test phase the overall group difference was significant (one-way ANOVA; *P* < 0.0007 and *P* < 0.0003 for protection and mitigation, respectively). No significant interaction between irradiation and 5-ITU treatment was noted for the NOR task (*P* = 0.323 and *P* = 0.133 for protection and mitigation, respectively; see Supplementary Tables). The unirradiated control mice showed a preference for the novel object, while irradiated mice showed a significantly reduced preference for novel object exploration (Fig. 2a,b; *P *<* *0.01). Irradiated mice that were treated with 5-ITU either prior to or after irradiation (20cGy + 5-ITU) exhibited significant improvements in object discrimination compared to the vehicle control irradiated mice (20cGy; *P *<* *0.05 and 0.01, respectively). The difference in DI between the control and 20cGy + 5-ITU cohorts of animals were statistically similar to each other and to the 0Gy + 5-ITU cohort. In all cases analysis of the time spent exploring the novel object as compared to the time spent exploring the familiar object indicate that 0Gy, 0Gy + 5-ITU and 20cGy + 5-ITU showed more novel object exploration behavior as compared with 20cGy animals.

Habituation and testing for the OiP task was initiated immediately after completion of the NOR test. As with the NOR task, the total exploration time for each object was not different during the familiarization phase between any of the four experimental groups. The overall group difference was significant for the radioprotection study during the OiP test phase (Fig. 2a; one-way ANOVA, *P* < 0.0001). Two-way ANOVA showed an interaction between radiation exposure and 5-ITU treatments for the protection study, but no significant interaction for the mitigation study in the OiP task (*P* = 0.035; see Supplementary Tables). In the 5-ITU radioprotection study, the irradiated mice showed a significantly reduced preference for novel object location as compared to both the 0Gy and 0Gy + 5-ITU groups of animals (Fig. 2a,b; *P *<* *0.001). Pre irradiation treatment with 5-ITU provided significant protection against the effects of irradiation on this hippocampal-dependent behavior task and these animals spent significantly more time exploring the object that had been moved to a novel location (Fig. 2a; 20cGy + 5-ITU; *P *<* *0.05). A similar trend for adverse effects of irradiation and 5-ITU amelioration of those effects was observed in the mitigation paradigm (Fig. 2b). The difference in DI between the 0Gy and 0Gy + 5-ITU cohorts of animals were statistically similar to each other. In all cases analysis of the time spent exploring the novel placement of objects as compared to the time spent exploring the same objects in their previous, familiar location indicate that irradiated animals (20cGy) spent less or equal time exploring novel object placement as compared to all other groups.

TO testing was initiated with no further habituation following completion of OiP task. During each familiarization phase, the exploration time for each object presented did not change between the four experimental groups. The overall group difference was significant during for the protection and mitigation studies (one-way ANOVA; *P* < 0.014 and *P* < 0.0001 for protection and mitigation, respectively). The two-way ANOVA revealed no significant interaction between irradiation and 5-ITU treatments for the TO task in the protection study (*P* = 0.101), whereas the interaction was significant for the mitigation study (*P* = 0.002) (see Supplementary Tables). In all cases, control mice exhibited better memory than the irradiated mice and spent more time exploring the first object they had seen in training phase I rather than the more recent object that they’d seen in training phase II (Fig. 2a,b; *P *<* *0.01). The mice that had received 5-ITU either pre- or post-irradiation (20cGy + 5-ITU) showed improved DI as compared to their respective concurrent control 20cGy cohorts, but that improvement was only statistically significant in the case of the mitigation study (Fig. 2b; *P *<* *0.001). In the protection study, and in the mitigation study as well, the difference in DI between the 0Gy and 0Gy + 5ITU cohorts of animals was not statistically different. In all cases analysis of the time spent exploring the less recently explored object as compared to the time spent exploring the more recently explored object indicate that 0Gy or animals treated with 5-ITU (0Gy + 5-ITU and 20cGy + 5-ITU) showed increased exploration of the less recently presented object as compared to the irradiated group (20cGy).

### ADK Protein Levels in the Irradiated Brain

**Figure 3:**
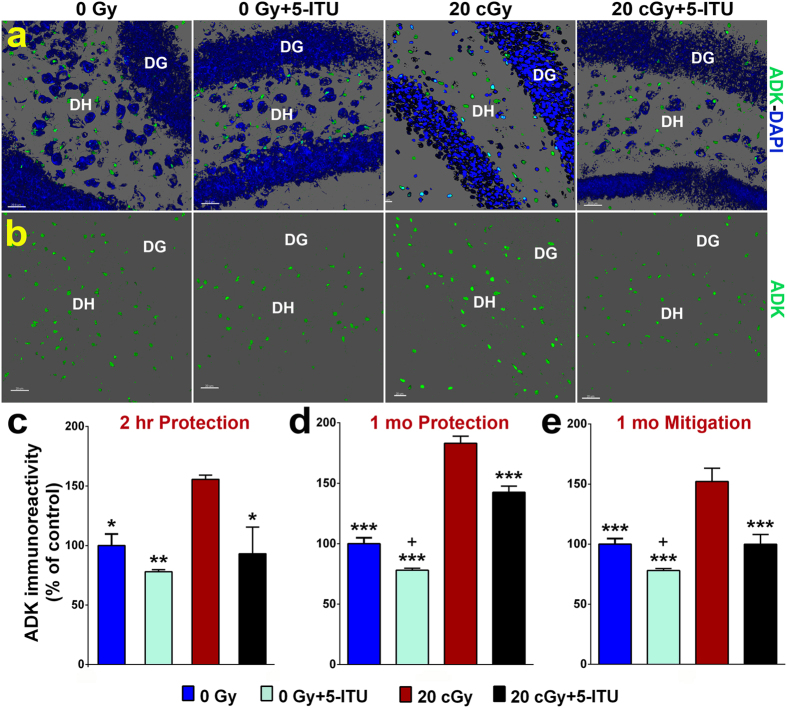
5-ITU treatment protects against ^28^Si particle irradiation induced increased ADK immunoreactivity. (**a**,**b**) Representative images illustrate that ADK protein levels are elevated 1 month post-irradiation (20cGy) that is reduced by 5-ITU treatment (ADK, green; DAPI nuclear counterstain, blue) in the hippocampal dentate hilus (DH) and dentate gyrus (DG). Deconvolution of images and quantification of ADK protein demonstrate that irradiation increased ADK at (**c**) 2 hrs post-irradiation and at (**d**,**e**) 1 month post-irradiation and that 5-ITU treatment protects against and mitigates this increase. Data are presented as mean ± SEM (N = 3–4 mice/group). *P* values are derived from ANOVA and Bonferroni’s multiple comparisons test (*P < 0.05, **P < 0.01, ***P < 0.001 for 0Gy, 0Gy + 5-ITU and 20cGy + 5-ITU as compared to 20cGy). ^+^*P* < 0.01 for 0Gy as compared to 0Gy + 5-ITU. (**a**,**b**) Scale bars 20 μm.

Following completion of the behavioral studies, the brains of these mice were evaluated for the effects of irradiation and 5-ITU treatments on epigenetic regulatory mechanisms. Immunohistochemistry and dual confocal microscopy were used to image and then semi-quantitatively measure the effect of ^28^Si particle irradiation on ADK protein levels in the hippocampal dentate gyrus (DG) (Fig. 3a,b). The overall group difference was significant for all experimental time points (one-way ANOVA, *P* < 0.0001). Radiation exposure induced a significant increase in ADK at 2 hours post-irradiation (Fig. 3c; *P *<* *0.05), and similarly, ADK protein levels were increased at 1 month post-irradiation (Fig. 3c,d; *P *<* *0.001). In the protection study, irradiated mice treated with 5-ITU prior to irradiation (20cGy + 5-ITU) had levels of ADK significantly lower than that observed in the 20cGy only mice at 2 hours post-irradiation and statistically indistinguishable from 0Gy controls (Fig. 3c; *P *<* *0.05). One month later, pre-irradiation 5-ITU treatments continued to protect mice against radiation-induced increases in ADK (Fig. 3d; *P *<* *0.001). In the mitigation study, post-irradiation 5-ITU treatments also reduced ADK protein levels 1 month post-irradiation (Fig. 3e; *P *<* *0.001). Two-way ANOVA indicated a significant interaction between irradiation and 5-ITU treatments for the 1-month mitigation study (*P* = 0.043; see Supplementary Tables). 0Gy + 5-ITU mice exhibited slightly lower levels of ADK protein as compared to the other three groups at all time points, although not always to a statistically significant level. Together these data suggest the effectiveness of 5-ITU treatments in modulating radiation-induced increases in ADK levels in the hippocampus. The observed pharmacologic control of ADK protein levels correlated closely with the protection against and mitigation of radiation induced behavioral deficits.

### Radiation Induced Changes in Global Levels of DNA Methylation

**Figure 4:**
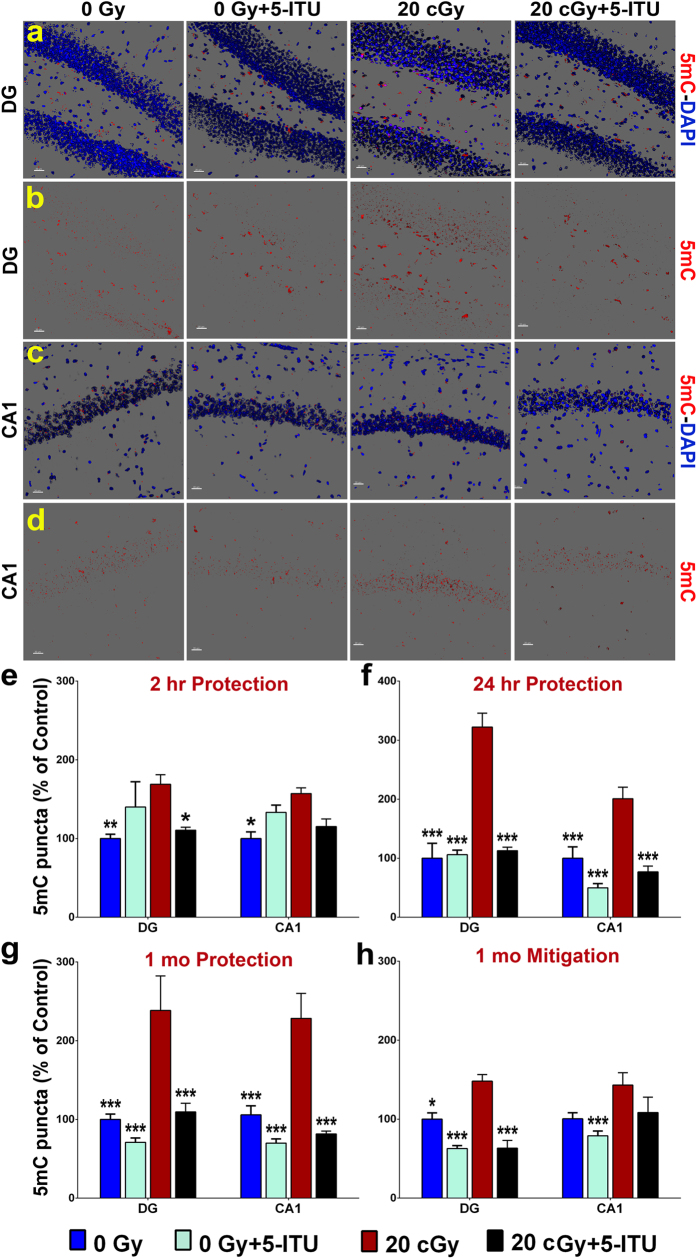
^28^Si particle irradiation increases levels of 5-methylcytosine in the hippocampus. Representative images show that radiation exposure causes increased 5mC levels in (**a**,**b**) DG and (**c**,**d**) CA1 regions of the brain and that is reduced by 5-ITU treatment (5mC, red; DAPI nuclear counterstain, blue). Deconvolution of images and quantification of 5mC demonstrate that irradiation induces increased 5mC at (**e**) 2 hrs, (**f**) 24 hrs and (**g**) 1 month post-irradiation and that 5-ITU treatment prior to irradiation protects against this increase. (**h**) Post-irradiation 5-ITU treatment also mitigates the radiation induced increase in 5mC. Data are presented as mean ± SEM (N = 3–4 mice/group). *P* values are derived from ANOVA and Bonferroni’s multiple comparisons test. *P < 0.05, **P < 0.01, ***P < 0.001 for 0Gy, 0Gy + 5-ITU and 20cGy + 5-ITU as compared to 20cGy. (**a–d**) Scale bars 20 μm.

To determine the effect of ^28^Si particle irradiation on global levels of DNA methylation in the hippocampus we used immunostaining for 5mC and analyzed deconvoluted confocal images (Fig. 4a–d). Global levels of 5mC were significantly elevated in both the DG and CA1 regions of the hippocampus of the irradiated mice. The overall group difference was significant at 2 hr and 24 hrs post-irradiation (one-way ANOVA; *P* < 0.05 and *P* < 0.001 for 2 hr and 24 hrs, respectively). While the effect of irradiation was relatively small at 2 hrs post exposure (Fig. 4e; *P *<* *0.01 and 0.05, for the DG and CA1 regions, respectively), the magnitude of increase was marked at 24 hrs post-irradiation (Fig. 4f; *P *<* *0.001 for both the DG and CA1). In the protection study, pre-irradiation 5-ITU treatment only modestly reduced 5mC levels at 2 hrs post-irradiation (Fig. 4e; 20cGy + 5-ITU; *P *<* *0.05 for the DG), but at 24 hrs post-irradiation the 20cGy + 5-ITU mice exhibited significantly reduced levels of 5mC as compared to the irradiated mice (Fig. 4f; *P *<* *0.001), that were statistically indistinguishable from control animals. The effect of ^28^Si particle irradiation on 5mC, persisted at 1 month post-irradiation in the protection study (significant overall difference, *P* < 0.001) and again the levels of 5mC in the 20cGy + 5-ITU mice was comparable to that of control mice (Fig. 4g; *P *<* *0.001). In the mitigation study, the radiation exposure effected much more subtle increases in 5mC levels (overall significant group difference, *P* < 0.001) that were only statistically significant in the DG region of the hippocampus (Fig. 4h; *P *<* *0.02), but in that instance the post-irradiation 5-ITU treatment significantly reduced 5mC levels relative to the 20cGy group of mice (*P *<* *0.001). Additional two-way ANOVA revealed an overall significant interaction between radiation exposure and 5-ITU treatments on the expression of 5mC (*P* = 0.05–0.001; see Supplementary Tables).

**Figure 5:**
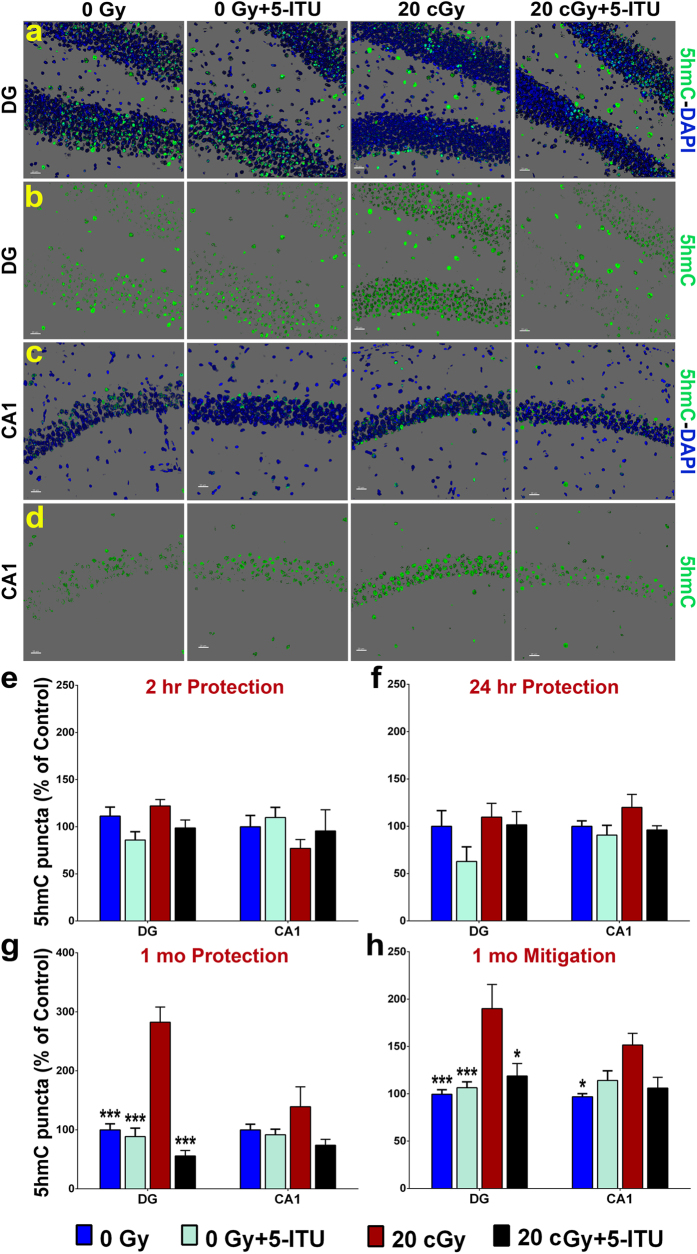
^28^Si particle irradiation causes increased levels of 5-hydroxymethylcytosine in the hippocampus 1 month post-irradiation. Representative images show that irradiation causes increased 5mC levels in the (**a**,**b**) DG and (**c**,**d**) CA1 regions of the brain and that is reduced by 5-ITU treatment (5hmC, green; DAPI nuclear counterstain, blue). Deconvolution of images and quantification of 5hmC demonstrate that 5hmC marks are not increased at (**e**) 2 hrs or (**f**) 24 hrs post-irradiation. Radiation exposure does cause elevated 5hmC at (**g**) 1 month post-irradiation and that 5-ITU treatment prior to irradiation provides some protection against this increase. (**h**) Post-irradiation 5-ITU treatment also provides some mitigation of this radiation effect. Data are presented as mean ± SEM (N = 3–4 mice/group). *P* values are derived from ANOVA and Bonferroni’s multiple comparisons test. *P < 0.05, **P < 0.01, ***P < 0.001 for 0Gy, 0Gy + 5-ITU and 20cGy + 5-ITU as compared to 20cGy. (**a**–**d**) Scale bars 20 μm.

5mC is actively converted to 5hmC by TET enzymes. The highest levels of 5hmC are found in the brain and recent evidence suggests that this epigenetic modification plays an active role in regulating brain function^18^. For this reason, in addition to measuring global levels of 5mC, we also measured global levels of 5hmC in the hippocampus of the irradiated mouse brain (Fig. 5a–d). Global levels of 5hmC were not affected by exposure to 20cGy ^28^Si particle irradiation at either 2 hrs or 24 hrs post-irradiation (Fig. 5e,f). In the protection study, increases in 5hmC were observed in the irradiated mice (20cGy) 1 month post exposure (with overall group difference, *P* < 0.01, one-way ANOVA), but only in the DG region of the hippocampus did those changes reach statistical significance (Fig. 5g; *P *<* *0.001). Mice that received the pre-irradiation treatment with 5-ITU (20cGy + 5-ITU) exhibited levels of 5hmC indistinguishable from that of 0Gy control animals (overall group difference, *P* < 0.001). In the mitigation study the irradiated mice had significant increases in 5hmC levels in both the DG and CA1 regions of the hippocampus relative to 0Gy controls (Fig. 5h; *P *<* *0.001 and *P *<* *0.05, respectively). The mice that received 5-ITU treatment post-irradiation exhibited reduced 5hmC levels, but only in the DG region of the hippocampus did those changes reach statistical significance (*P *<* *0.05). Overall, additional two-way ANOVA did not indicate any interaction between irradiation and 5-ITU treatment on the immunoreactivity of 5hmC (see Supplementary Tables).

Together, these data suggest that radiation exposure causes an acute epigenetic response with a marked increase in the accumulation of 5mC 2 hrs post-irradiation — an effect that is also evident at 1 month post-irradiation. A lack of early post-irradiation changes in global 5hmC levels was observed, however the changes in 5hmC that were observed 1 month post-irradiation, coincident with behavioral impairments, may represent part of a delayed epigenetic radiation response.

### Effect of Irradiation on DNA Methylating Enzymes

**Figure 6:**
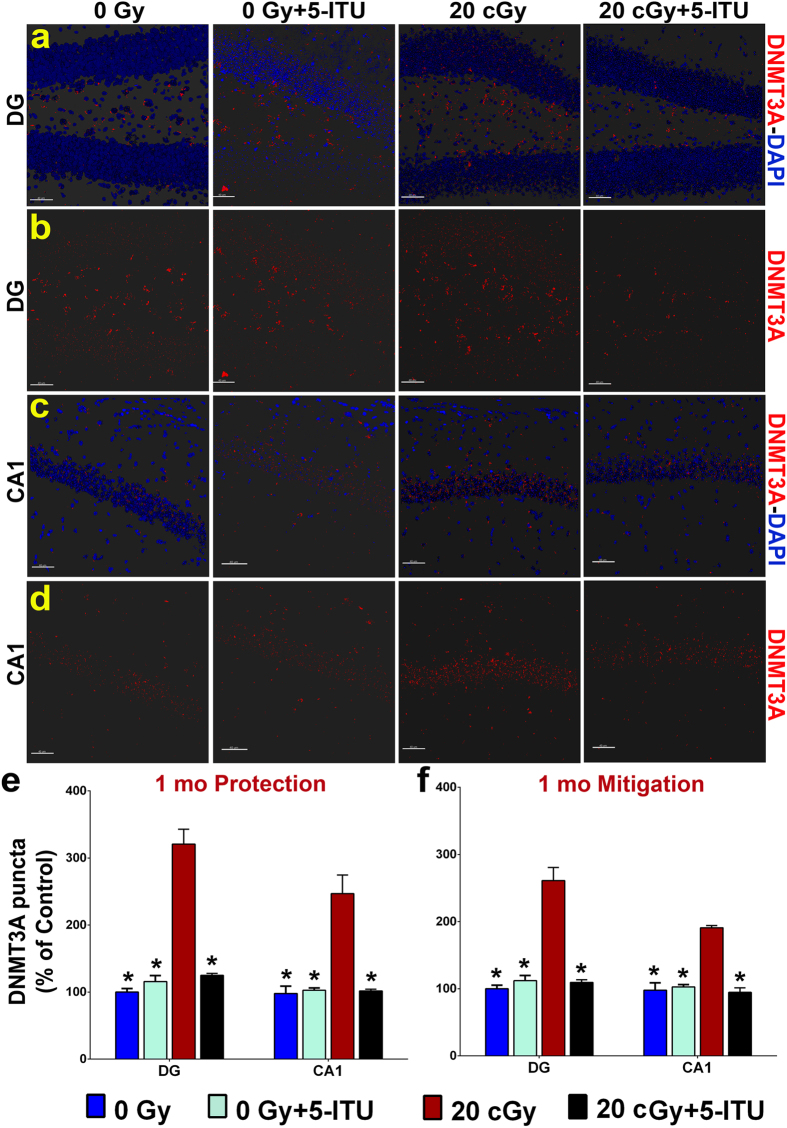
^28^Si particle irradiation increases levels of DNMT3a protein in the hippocampus. Representative images show that irradiation causes increased levels of DNMT3a in (**a**,**b**) DG and (**c**,**d**) CA1 regions of the brain and that is reduced by 5-ITU treatment (DNMT3a, red; DAPI nuclear counterstain, blue). Deconvolution of images and quantification of DNMT3a demonstrate that radiation exposure causes elevated DNMT3a at 1 month post-irradiation and that 5-ITU treatment (**e**) prior to irradiation provides protection against this increase and that 5-ITU treatment (**f**) after irradiation mitigates this radiation effect. Data are presented as mean ± SEM (N = 3–4 mice/group). *P* values are derived from ANOVA and Bonferroni’s multiple comparisons test. *P < 0.05, **P < 0.01, ***P < 0.001 for 0Gy, 0Gy + 5-ITU and 20cGy + 5-ITU as compared to 20cGy. (**a**–**d**) Scale bars 40 μm.

Protein levels of DNMT3a, a *de novo* DNA methyltransferase, were evaluated 1 month post-irradiation (Fig. 6a–d). The overall group difference was significant for both the radioprotection and for the mitigation studies (one-way ANOVA, *P* < 0.0001). Exposure to 20cGy of ^28^Si particles induced significant increases in global levels of DNMT3a as compared to each group’s respective control cohort (Fig. 6e,f; *P* < 0.0001), and mice that received either the pre-irradiation or post-irradiation treatment with 5-ITU (20cGy + 5-ITU) exhibited DNMT3a levels that were indistinguishable from that of 0Gy controls. These data correlate with both the effects of exposure on cognition and on the levels of 5mC in the irradiated brain at this same 1 month time point. Additionally, the interaction between irradiation and 5-ITU treatment on the DNMT3a levels in the DG and CA1 subfields were significant (two-way ANOVA, *P* < 0.0001; see Supplementary Tables).

**Figure 7:**
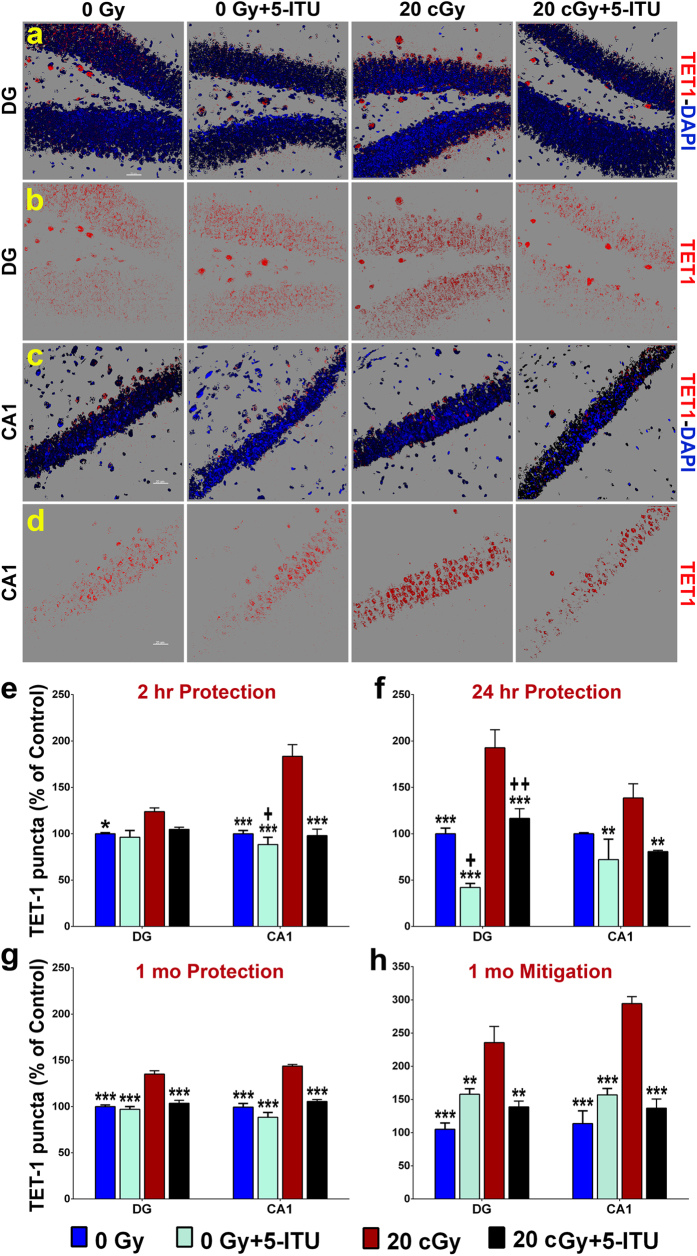
^28^Si particle irradiation increases levels of TET1 protein in the hippocampus. Representative images show that radiation exposure causes increased TET1 levels in (**a**,**b**) DG and (**c**,**d**) CA1 regions of the brain and that is reduced by 5-ITU treatment (TET1, red; DAPI nuclear counterstain, blue). Deconvolution of images and quantification of TET1 demonstrate that irradiation induces increased protein levels at (**e**) 2 hrs, (**f**) 24 hrs and (**g**) 1 month post-irradiation and that 5-ITU treatment prior to irradiation protects against this increase. (**h**) Post-irradiation 5-ITU treatment also mitigates the radiation induced increase in TET1. Data are presented as mean ± SEM (N = 3–4 mice/group). *P* values are derived from ANOVA and Bonferroni’s multiple comparisons test. *P < 0.05, **P < 0.01, ***P < 0.001 for 0Gy, 0Gy + 5-ITU and 20cGy + 5-ITU as compared to 20cGy. ^+^*P* < 0.01 ^++^*P* < 0.002 for 0Gy + 5-ITU as compared to 20cGy + 5-ITU. (**a**–**d**) Scale bars 20 μm.

**Figure 8:**
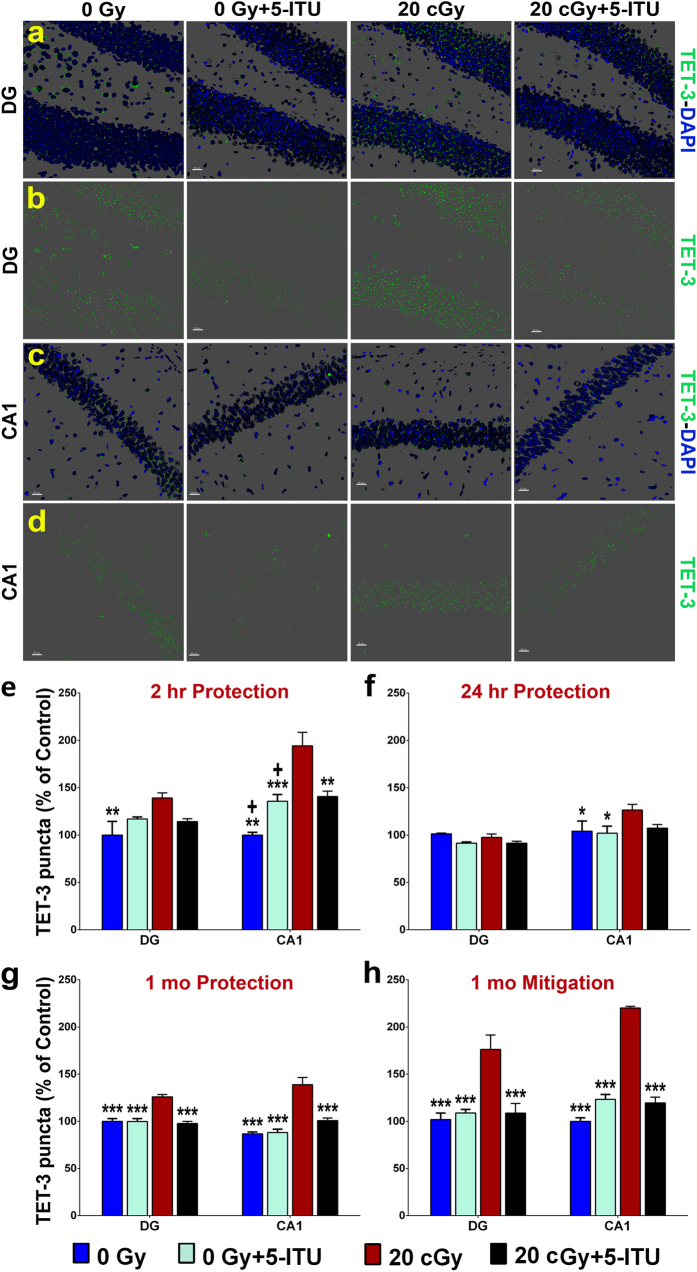
^28^Si particle irradiation causes increased levels of TET3 protein in the hippocampus. Representative images show that radiation exposure causes increased TET3 levels in (**a**,**b**) DG and (**c**,**d**) CA1 regions of the brain and that is reduced by 5-ITU treatment (TET1, green; DAPI nuclear counterstain, blue). Deconvolution of images and quantification of TET3 demonstrate that irradiation induces increased protein levels at (**e**) 2 hrs, (**f**) 24 hrs and (**g**) 1 month post-irradiation and that 5-ITU treatment prior to irradiation protects against this increase. (**h**) Post-irradiation 5-ITU treatment also mitigates the radiation induced increase in TET3. Data are presented as mean ± SEM (N = 3–4 mice/group). *P* values are derived from ANOVA and Bonferroni’s multiple comparisons test. *P < 0.05, **P < 0.01, ***P < 0.001 for 0Gy, 0Gy + 5-ITU and 20cGy + 5-ITU as compared to 20cGy. ^+^*P* < 0.02 for 0Gy as compared to 0Gy + 5-ITU 0Gy + 5-ITU and for 0Gy as compared to 20cGy + 5-ITU. (**a–d**) Scale bars 20 μm.

TET1 and TET3 proteins convert 5mC to 5hmC via oxidation of 5mC. Increased levels of these enzymes in the irradiated brain would support the hypothesis that the increased 5hmC is part of an active epigenetic response to radiation exposure. Therefore we evaluated not only DNMT3a, but also the global levels of the TET proteins, again using confocal analyses of immunohistochemically labeled tissues (Figs 7a–d and 8a–d). The overall group difference was significant at 2 hr post-irradiation for both TET proteins (one-way ANOVA, *P* < 0.02 and *P* < 0.0001 for TET1 and TET3, respectively). Two hours post-irradiation we observed increased TET1 and TET3 protein level in the DG and CA1 regions of the hippocampus of the 20cGy mice as compared to controls (Figs 7e and 8e; *P *<* *0.05 and 0.001 and *P *<* *0.01 and 0.01 for each region of the brain for TET1 and TET3, respectively). In both cases, the effect was more modest in the DG as compare to the CA1 and 5-ITU was only able to significantly modulate this increase in the CA1 (*P *<* *0.001 and *P *<* *0.01 for TET1 and TET3, respectively). When TET1 and TET3 were evaluated 24 hrs post-irradiation significant increases were observed only for TET1 (significant overall group difference, *P* < 0.001) in the DG and for TET3 in the CA1 (Figs 7f and 8f; *P *<* *0.001 and *P *<* *0.05, respectively compared to 20cGy). 5-ITU treatment effectively protected against the increase in TET1 in both the DG and CA1 for the 20cGy + 5-ITU animals (Fig. 7f; *P *<* *0.001 and 0.01, respectively).

At 1 month post-irradiation, TET1 and TET3 were significantly increased in both regions of the hippocampus in the protection study (Figs 7g and 8g; *P *<* *0.001). This radiation response was similarly observed for both proteins in the mitigation study (Figs 6h and 7h; *P *<* *0.001). The overall group difference was significant for TET1 and TET3 (*P* < 0.001). Further, in both the protection and mitigation studies the irradiated mice that were treated with 5-ITU either pre- or post-irradiation had significant reductions in TET1 and TET3 protein levels. For the protection study, the TET1 and TET3 reductions reached levels of significance of *P *<* *0.001 for all measurements (Figs 7g and 8g). For the mitigation study, the TET1 reductions reached levels of significance of *P *<* *0.01 for the DG and *P *<* *0.001 for the CA1 (Fig. 7g), and TET3 reductions reached levels of significance *P *<* *0.001 in all cases (Fig. 8g). In almost all instances, mice that received the pre-irradiation or post-irradiation treatments with 5-ITU (20cGy + 5-ITU) exhibited levels of TET1 and TET3 protein that were indistinguishable from that of 0Gy control animals 1 month post exposure. The fact that TET1 and TET3 protein levels are inconsistently affected by radiation exposure at 2 and 24 hr correlates with the unaltered levels of 5hmC at those same times and suggest lag time between the radiation induced increases in 5mC and TET mediated increases in 5hmC. Overall, two-way ANOVA revealed significant interactions between radiation exposure and 5-ITU treatment 1 month later on the expression of TET1 and TET3 in the DG and CA1 subfields of the hippocampus (*P* < 0.001; see Supplementary Tables).

## Discussion

The results of this study are unique, establishing the possibility that neuroepigenetic mechanisms significantly contribute to the functional and structural changes that affect the irradiated brain and cognition. The exposed mice had significantly elevated levels of 5mC and 5hmC that correlated with an impaired ability to perform behavioral tasks that measure episodic, spatial and temporal memory, and that are dependent on hippocampal, as well as cortical, function. 5-ITU treatment mediated protection against and mitigation of this radiation response. While we currently cannot exclude non-specific effects of 5-ITU treatment our data indicate that 5-ITU effected significant changes global DNA methylation in the irradiated brain, and improved cognition in those same animals.

Generally, DNA methylation has been considered to be a stable modification, particularly in non-dividing, differentiated cells such as neurons. However, studies have now shown that DNA methylation dynamically changes as part of learning and memory formation, and the response to external stimuli^6^,^7^,^8^,^22^,^25^. Based on location and morphology (dentate gyrus blades and CA1 pyramidal layers), the results of our study suggest that the increases in 5mC and 5hmC that we observe post-irradiation also occur primarily in neurons. The increase in 5mC is detectable within 2 hours of irradiation, suggesting a rapid epigenetic response that is persistent given the significant increase seen one month later. However, we observed no increase in 5hmC at 2 or 24 hours post-irradiation and modest or inconsistent increase in both TET1 and TET3 proteins in the irradiated brain at those same early post-irradiation times. Subsequent work using methylated DNA immunoprecipitation assays (MeDIP or 5hmC-seq) and qRT-PCR will determine whether the observed radiation-induced changes in DNA methylation functionally alter the expression of genes involved in CNS function.

The observed time course suggests that more time is required for increases in the 5hmC cytosine modification be effected and reach measurable levels of change. Given that both TET1 and TET3 are elevated one month post-irradiation, when we also see significant increases in global 5hmC, it is unclear whether one, the other, or both TET enzymes are key to the modification of 5mC to 5hmC. However, it has been suggested that TET1 overexpression in the adult hippocampal DG leads to increased global levels of 5hmC^6^,^7^ and TET1 overexpression in the CA1 region of the hippocampus has been shown to induce deficits in contextual fear conditioning^17^,^21^.

Among the cells of the brain, DNMT1 and DNMT3a are the most active in neurons. While DNMT1 is broadly considered a maintenance methyltransferase and DNMT3a is considered a *de novo* methyltransferase, overlap has been shown in their functions^23^. DNMT1 and DNMT3a levels remain high in post mitotic neurons, implying a specific role for these enzymes in the adult brain that goes beyond the classic view of *de novo* and maintenance DNMT. Our data do not preclude the involvement of DNMT1 in the radiation response. In fact the double, but not the single, knock-out (KO) mouse model of *Dnmt3a* and *Dnmt1* in forebrain excitatory neurons shows impairment in hippocampal plasticity and learning and memory^14^, suggesting coordinated or redundant activity of these DNMT in mature neurons. Furthermore, DNMT1 and DNMT3a activity has been shown to foster neuronal plasticity in the adult in the context of fear-related memory^8^ (Miller 2010) and age related cognitive decline^37^. Forthcoming studies from our laboratory using ChIP-sequencing will determine whether the immunohistochemical changes that we observe are reflected in DNMT and TET protein occupancy to affect gene expression.

While the mechanisms by which 5-ITU might cause the modulation of DNMT3a and TET protein levels observed in our study is not immediately apparent, it may be driven in part by the levels of 5mC substrate available to these enzymes. Alternatively, expression of these genes is also likely to be under epigenetic control and changes in DNA methylation of regulatory elements for them drives the changes in protein levels. The latter explanation might be a plausible answer at the delayed time points, but not as plausible at two hours post-irradiation.

The neuroepigenetic systems that regulate brain function are exceedingly complex and our understanding of the role for these molecular mechanisms in the CNS will continue to evolve. While impairments quantified on our selected spontaneous exploration tasks are likely due to multiple underlying causes, alterations in functional connectivity could explain much of our findings. Interpretation of behavioral data will always involve some inherent inter-individual variability, which may explain differences in overall exploration times between select cohorts or animals. Despite these and other caveats, cosmic radiation exposure seems to have altered the capability of an animal to perceive and respond to a given set of specific sensory information, a process that our data suggest may be effected by upstream epigenetic regulation. By interrogating the connectivity of other regions of the brain using learning and memory tasks that are less reliant on movement and exploration we hope to further clarify our observations. In the context of the proposed role for epigenetics in the function of the irradiated brain, future studies will determine more specifically how changes in DNA methylation alter radiation induced cognitive dysfunction. It will also be important to determine the differences between locus specific DNA methylation marks and global levels of 5mC and 5hmC. It is unclear whether global changes in DNA methylation and histone modifications affect chromatin structure to alter gene expression and cellular phenotype or gene specific DNA methylation. Similarly, until recently it was generally accepted that cytosine methylation was confined to CpG dinucleotide sequences in the mammalian genome. Now, DNA methylation is being observed in non-CpG contexts in the brains of adult mice and humans that may play a defining role in neural or glia specific functions^38^,^39^. Nevertheless, our data support the hypothesis that neuroepigenetic aberrations contribute to cognitive deficits following space relevant radiation exposures, and inhibition of that radiation-induced hypermethylation protects against and mitigates those effects.

## Materials and Methods

### Animals, drug treatments, and irradiations

All animal experimentation procedures described in this study are in accordance with the guidelines provided by NIH and approved by the University of California Irvine and Brookhaven National Laboratory Institutional Animal Care and Use Committees. Animals were maintained in standard housing conditions (20 °C ± 1 °C; 70% ± 10% humidity; 12 h:12 h light and dark cycle) and provided ad libitum access to standard rodent chow and water. For each of the separately performed independent studies (protection or mitigation), single cohorts of 6 month old wild type male mice (C57Bl6/J, Jackson Laboratory, Bar Harbor ME) were stratified by body weight and randomly assigned to each of four experimental groups (0Gy, 0Gy + 5-ITU, 20cGy, 20cGy + 5-ITU). Behavioral studies included N = 8–10 mice per group. After behavior studies were completed, immunohistochemical analyses were performed on a subset of the same mice that had been used in the behavior studies (approximately 6 wks post-irradiation; N = 3–4 mice/group). For the protection study an additional 3–4 mice were included in each group for evaluation of molecular endpoints at 2 hrs and at 24 hrs post-irradiation for a total N = 16–20 mice in each of the four experimental groups. Mice were acclimatized for 3–7 days prior to initiation of the study at the NASA Space Radiation Laboratory (NSRL), Brookhaven National Laboratory (BNL, Upton, NY).

The ADK inhibitor 5-ITU (5-iodotubercidin; HY-15424, NSC 113939, MedChem Express, Princeton, NJ, USA) was prepared fresh daily by dissolving in saline with 2% ethanol (v/v; Sigma, St. Louis, MO). Animals received either vehicle (2% ethanol in saline) or 5-ITU (1.6 mg/kg) via intraperitoneal (i.p) injection for 6 days prior to irradiation (protection) or for 6 days post-irradiation (mitigation). The 5-ITU dose was based on previous studies^28^,^33^ and neither drug treatment nor irradiation resulted in a change in body weight of the mice.

During irradiation mice were loosely restrained in Lucite containers with breathing holes (3 in. × 1.5 in. × 1.5 in.) for exposure to 20cGy of 600 MeV/n ^28^Si particles (dose rate = ~20cGy/min; LET = ~54 keV/μm) in experimental cycles NSRL14C for the protection study and NSRL15B for the mitigation study. The Physics Dosimetry Group of the NSRL provided beam characterization and dosimetry. Concurrent control mice were placed in Lucite boxes at the NSRL for the same length of restraint time as required for the radiation exposures.

### Behavioral testing

To determine the effect of altered DNA methylation on cognitive function after irradiation, mice were subject to behavioral testing 1 month after irradiation. Testing occurred over 2 weeks and included 3 open field, spontaneous exploration tasks in the following order; novel object recognition (NOR), object in place (OiP), and temporal order (TO). These tasks rely on intact hippocampus, medial prefrontal cortex (mPFC) and perirhinal cortex function. The NOR task evaluates the preference for novelty, the OiP task evaluates associative recognition memory and the TO task provides a measure of temporal order memory. Tasks were conducted as described previously^2^ and all trials were scored by an observer blind to the experimental groups in order to avoid introducing bias into the data collection process. The average of those scores was used to determine performance defined as a discrimination index (DI) and calculated as [(novel location exploration time/total exploration time) – (familiar location exploration time/total exploration time)] × 100.

### Immunohistochemistry

After completion of behavioral testing, mice were deeply anesthetized using isoflurane and euthanized via intercardiac perfusion using 4% paraformaldehyde (ACROS Organics; NJ) in 100 mM phosphate buffered saline (PBS; pH 7.4, Gibco). Brains were cryoprotected using a sucrose gradient (10–30%) and sectioned coronally into 30 μm thick sections using a cryostat (Leica Microsystems, Germany). For each endpoint 3–4 representative coronal brain sections from each of 3–4 animals per experimental group were selected at approximately 15 section intervals to encompass the rostro-caudal axis from the middle of the hippocampus (ranging from AP −2.1 to −2.95 from Bregma) and stored in tris buffered saline (TBS, 100 mM, pH 7.4, Sigma-Aldrich, St. Louis, MO). The free floating sections were first rinsed in TBS followed by Tris-A (TBS with 0.1% Triton-X-100, Sigma), blocked with 10% normal goat serum (NGS with Tris-A, Sigma) and incubated overnight in rabbit anti-ADK (1:3000; Bethyl Laboratories, Montgomery, TX) antibody prepared in 3% normal goat serum (NGS) and Tris-A. The next day, the sections were treated with goat anti-rabbit Alexa Fluor 488 (1:750; Life Technologies/Invitrogen) made with Tris-A and 3% NGS. The sections were nuclear counterstained with DAPI (1 μmol/L in TBS, 15 min). Similarly, for the immunofluorescence of 5mC and 5hmC, mouse monoclonal anti-5mC (1:2000; Epigentek; Farmingdale, NY) and rabbit polyclonal anti-5hmC (1:5000; Active Motif, Carlsbad, CA) primary antibodies were used with Alexa Fluor 594 and Alexa Fluor 488 secondary antibodies, respectively, at dilutions of 1:750. For the immunofluorescence of DNMT3a, mouse monoclonal anti-DNMT3a primary antibody (1:200; Novus Biologicals, Littleton, CO) was used with Alexa Fluor 594 secondary antibody at a dilution of 1:500. For the immunofluorescence of TET1 and TET3 rabbit polyclonal primary antibodies were used at dilutions of 1:200 and 1:500, respectively (Abcam; Cambridge, MA) with Alexa Fluor 594 and Alexa Fluor 488 secondary antibodies, respectively, at dilutions of 1:500.

### Confocal microscopy, image processing and 3D quantification

The dual immunostained coronal brain sections were scanned using a confocal microscope (Nikon Eclipse Ti C2) equipped with a 40× PlanApo oil-immersion lens (1.3 NA, Nikon) and an NIS-Elements AR interface (v4.30, Nikon). 30 z stacks (1024 bit depth) at 0.5 μm from three different fields (318 × 318 × 24 μm) in each section were imaged from the dentate gyrus and from the CA1 subfields. 5hmC, TET-3 and ADK immunofluorescence was imaged with 493 nm excitation and 518 nm emission and 5mC, DNMT3A and TET-1 immunofluorescence was imaged with 592 nm excitation and 617 nm emission. The digitized z stacks were deconvoluted using the AutoQuant software (version X3.0.4, Media Cybernetics, Rockville, MD) with 1.26867 × 1.26867 × 1 μm spacing, and wavelengths set at 447 nm (DAPI), 510 (5hmC, TET-3, ADK) and 594 nm (5mC, DNMT3A, TET-1). An adaptive, 3D blinded method was used to create deconvoluted images for direct import into the Imaris module (version 8.1.2, Bitplane, Inc., Zurich, Switzerland). The 3D algorithm-based surface rendering and quantification of fluorescence intensity for each fluorescently labeled marker was carried out in Imaris at 100% rendering quality. Each channel was analyzed separately. 3D surface rendering detects immunostained puncta or nuclear staining (DAPI) satisfying pre-defined criteria, for the puncta size (0.5 to 1 μm) verified visually for accuracy. Using deconvoluted confocal z stack volume from the control group (untreated, unirradiated) as a baseline for the minimum thresholding, a channel mean intensity filter was applied and used for all the experimental groups for each batch of molecular markers. The pre-set parameters were kept constant throughout the subsequent analysis of immunoreactivity for each antigen. To maintain uniformity among the varying number of puncta for each individual time point and/or antigen analyzed, the number of puncta per 318 × 318 × 24 μm was normalized to control and data was expressed as a mean immunoreactivity (percentage) relative to unirradiated controls.

### Statistical Analyses

Statistical analyses were carried out using GraphPad Prism (v6). One-way analysis of variance (ANOVA) was used to assess the normal distribution of data and the significance between control and irradiated groups of mice receiving vehicle control or 5-ITU treatment. When overall group effects were found to be statistically significant, the Bonferroni multiple comparisons test was used to reveal the effect of 5-ITU treatment and irradiation in each experimental group. Additionally, two-way ANOVA was used to assess the interaction of irradiation and 5-ITU treatments on the behavioral and molecular parameters. A *P* value of <0.05 was considered to be statistically significant. All one-way and two-way ANOVA data is provided in the Supplementary Information section.

## Additional Information

**How to cite this article:** Acharya, M. M. *et al*. Epigenetic determinants of space radiation-induced cognitive dysfunction. *Sci. Rep.* **7**, 42885; doi: 10.1038/srep42885 (2017).

**Publisher's note:** Springer Nature remains neutral with regard to jurisdictional claims in published maps and institutional affiliations.

## Supplementary Material

Supplementary Information
