# Supplementary material for: Epigenetic determinants of space radiation-induced cognitive dysfunction
Source: Sci Rep. 2017 Feb 21;7:42885. doi: 10.1038/srep42885 (PMC5318883; doi:10.1038/srep42885)
Supplement: Supplementary Information [file srep42885-s1.doc]

**Supplementary Information**

**Epigenetic determinants of space radiation-induced cognitive dysfunction**

Munjal M. Acharya, Al Anoud D. Baddour, Takumi Kawashita, Barrett D. Allen,

Amber R. Syage, Thuan H. Nguyen, Nicole Yoon, Erich Giedzinski, Liping Yu,

Vipan K. Parihar, and Janet E. Baulch^1^

University of California Irvine, CA 92697, USA

^1^Corresponding author:

Dr. Janet E. Baulch

Department of Radiation Oncology

University of California Irvine

Medical Sciences I, Room B-146D

Irvine CA 92697-2695

Phone: (949) 824-7396

Fax: (949) 824-3566

E-mail: [jbaulch@uci.edu](mailto:jbaulch@uci.edu)

**Supplemental Information 1:** Exploration time during novel object recognition (NOR), object in place (OiP) and temporal order (TO) tasks. Control mice displayed preference for exploring the novel object or placement of objects over the familiar object or spatial location that was not shown by irradiated mice. In contrast, irradiated mice receiving 5-ITU showed improvements in preference towards a novel object or place. Data are presented as mean + SEM (N = 8-10 mice/group). See Methods for groups and behavioral testing protocol details.

| **Novel Object Recognition Task** | **Time spent exploring novel object**  **(sec)** | **Time spent exploring familiar object (sec)** | **Total exploration time**  **(sec)** |
| --- | --- | --- | --- |
| 0 Gy  (protection group) | 17.61 ± 4.02 | 9.38 ± 2.08 | 26.99 ± 5.85 |
| 0 Gy + 5-ITU  (protection / mitigation group) | 9.58 ± 3.53 | 3.57 ± 0.75 | 13.38 ± 4.60 |
| 20 cGy  (protection group) | 7.62 ± 1.99 | 9.43 ± 1.90 | 15.69 ± 4.29 |
| 20 cGy + 5-ITU  (protection group) | 5.75 ± 1.47 | 3.32 ± 1.11 | 8.06 ± 2.35 |
| 0 Gy  (mitigation group) | 7.68 ± 1.89 | 3.77 ± 0.71 | 13.16 ± 3.05 |
| 20 cGy  (mitigation group) | 15.60 ± 3.13 | 14.57 ± 2.19 | 30.17 ± 5.04 |
| 20 cGy + 5-ITU  (mitigation group) | 19.78 ± 1.72 | 11.18 ± 1.33 | 30.96 ± 2.94 |

| **Object in Place Task** | **Time spent exploring novel object location (sec)** | **Time spent exploring familiar location**  **(sec)** | **Total exploration time**  **(sec)** |
| --- | --- | --- | --- |
| 0 Gy  (protection group) | 24.37 ± 7.39 | 16.76 ± 5.77 | 41.13 ± 12.96 |
| 0 Gy + 5-ITU  (protection / mitigation group) | 22.63 ± 2.94 | 13.35 ± 1.70 | 35.99 ± 4.17 |
| 20 cGy  (protection group) | 16.07 ± 4.06 | 22.14 ± 5.56 | 38.21 ± 9.35 |
| 20 cGy + 5-ITU  (protection group) | 18.23 ± 2.18 | 16.58 ± 3.24 | 34.81 ± 5.42 |
| 0 Gy  (mitigation group) | 9.27 ± 0.76 | 6.41 ± 0.61 | 15.68 ± 1.16 |
| 20 cGy  (mitigation group) | 8.45 ± 0.85 | 7.44 ± 0.68 | 15.89 ± 1.44 |
| 20 cGy + 5-ITU  (mitigation group) | 8.04 ± 0.42 | 5.51 ± 0.12 | 13.21 ± 0.45 |

| **Temporal Order Task** | **Time spent exploring less recent object**  **(sec)** | **Time spent exploring recent object**  **(sec)** | **Total exploration time**  **(sec)** |
| --- | --- | --- | --- |
| 0 Gy  (protection group) | 10.90 ± 3.33 | 8.11 ± 2.49 | 19.01 ± 4.81 |
| 0 Gy + 5-ITU  (protection / mitigation group) | 10.00 ± 2.17 | 7.81 ± 1.26 | 17.81 ± 3.17 |
| 20 cGy  (protection group) | 6.10 ± 1.82 | 7.62 ± 2.13 | 13.71 ± 3.42 |
| 20 cGy + 5-ITU  (protection group) | 11.40 ± 3.45 | 8.44 ± 1.69 | 19.84 ± 5.13 |
| 0 Gy  (mitigation group) | 4.35 ± 0.86 | 3.35 ± 0.56 | 7.49 ± 1.39 |
| 20 cGy  (mitigation group) | 4.11 ± 0.51 | 4.88 ± 0.39 | 9.39 ± 0.56 |
| 20 cGy + 5-ITU  (mitigation group) | 6.62 ± 1.13 | 4.31 ± 0.95 | 10.30 ± 0.64 |

**Supplemental Information 2:** **Two-way ANOVA Tables.** See Methods for groups and behavioral testing and immunohistochemical staining protocol details (DF, the degrees of freedom in the source; SS, the sum of squares due to the source; MS, the mean sum of squares due to the source; F, the F-statistic).

NOR Protection

| **ANOVA table** | **SS** | **DF** | **MS** | **F (DFn, DFd)** | **P value** |
| --- | --- | --- | --- | --- | --- |
| Interaction | 209.8 | 1 | 209.8 | F (1, 20) = 1.030 | P = 0.3223 |
| IRR | 3086 | 1 | 3086 | F (1, 20) = 15.15 | P = 0.0009 |
| 5-ITU | 1973 | 1 | 1973 | F (1, 20) = 9.685 | P = 0.0055 |
| Residual | 4075 | 20 | 203.7 |  |  |

NOR Mitigation

| **ANOVA table** | **SS** | **DF** | **MS** | **F (DFn, DFd)** | **P value** |
| --- | --- | --- | --- | --- | --- |
| Interaction | 616.9 | 1 | 616.9 | F (1, 38) = 2.346 | P = 0.1339 |
| IRR | 3034 | 1 | 3034 | F (1, 38) = 11.54 | P = 0.0016 |
| 5-ITU | 2897 | 1 | 2897 | F (1, 38) = 11.02 | P = 0.0020 |
| Residual | 9992 | 38 | 262.9 |  |  |

**Oi**P Protection

| **ANOVA table** | **SS** | **DF** | **MS** | **F (DFn, DFd)** | **P value** |
| --- | --- | --- | --- | --- | --- |
| Interaction | 375.3 | 1 | 375.3 | F (1, 15) = 5.389 | P = 0.0348 |
| IRR | 2332 | 1 | 2332 | F (1, 15) = 33.49 | P < 0.0001 |
| 5-ITU | 603.5 | 1 | 603.5 | F (1, 15) = 8.666 | P = 0.0101 |
| Residual | 1045 | 15 | 69.64 |  |  |

**Oi**P Mitigation

| **ANOVA table** | **SS** | **DF** | **MS** | **F (DFn, DFd)** | **P value** |
| --- | --- | --- | --- | --- | --- |
| Interaction | 157.4 | 1 | 157.4 | F (1, 30) = 0.5836 | P = 0.4509 |
| IRR | 97.96 | 1 | 97.96 | F (1, 30) = 0.3633 | P = 0.5512 |
| 5-ITU | 477.2 | 1 | 477.2 | F (1, 30) = 1.770 | P = 0.1934 |
| Residual | 8090 | 30 | 269.7 |  |  |

TO Protection

| **ANOVA table** | **SS** | **DF** | **MS** | **F (DFn, DFd)** | **P value** |
| --- | --- | --- | --- | --- | --- |
| Interaction | 532.1 | 1 | 532.1 | F (1, 20) = 2.958 | P = 0.1009 |
| IRR | 1410 | 1 | 1410 | F (1, 20) = 7.840 | P = 0.0111 |
| 5-ITU | 767.2 | 1 | 767.2 | F (1, 20) = 4.266 | P = 0.0521 |
| Residual | 3597 | 20 | 179.8 |  |  |

TO Mitigation

| **ANOVA table** | **SS** | **DF** | **MS** | **F (DFn, DFd)** | **P value** |
| --- | --- | --- | --- | --- | --- |
| Interaction | 3013 | 1 | 3013 | F (1, 58) = 10.46 | P = 0.0020 |
| IRR | 110.4 | 1 | 110.4 | F (1, 58) = 0.3833 | P = 0.5383 |
| 5-ITU | 1987 | 1 | 1987 | F (1, 58) = 6.896 | P = 0.0110 |
| Residual | 16713 | 58 | 288.2 |  |  |

ADK 2h protection:

| **ANOVA table** | **SS** | **DF** | **MS** | **F (DFn, DFd)** | **P value** |
| --- | --- | --- | --- | --- | --- |
| Interaction | 1670 | 1 | 1670 | F (1, 13) = 1.919 | P = 0.1893 |
| IRR | 5083 | 1 | 5083 | F (1, 13) = 5.839 | P = 0.0311 |
| 5-ITU | 7254 | 1 | 7254 | F (1, 13) = 8.334 | P = 0.0127 |
| Residual | 11315 | 13 | 870.4 |  |  |

ADK 1mo protection:

| **ANOVA table** | **SS** | **DF** | **MS** | **F (DFn, DFd)** | **P value** |
| --- | --- | --- | --- | --- | --- |
| Interaction | 578.6 | 1 | 578.6 | F (1, 20) = 3.315 | P = 0.0836 |
| IRR | 32202 | 1 | 32202 | F (1, 20) = 184.5 | P < 0.0001 |
| 5-ITU | 5662 | 1 | 5662 | F (1, 20) = 32.45 | P < 0.0001 |
| Residual | 3490 | 20 | 174.5 |  |  |

ADK 1mo mitigation:

| **ANOVA table** | **SS** | **DF** | **MS** | **F (DFn, DFd)** | **P value** |
| --- | --- | --- | --- | --- | --- |
| Interaction | 1384 | 1 | 1384 | F (1, 19) = 4.682 | P = 0.0434 |
| IRR | 7531 | 1 | 7531 | F (1, 19) = 25.47 | P < 0.0001 |
| 5-ITU | 7540 | 1 | 7540 | F (1, 19) = 25.50 | P < 0.0001 |
| Residual | 5618 | 19 | 295.7 |  |  |

5mC 2h protection: DG

| **ANOVA table** | **SS** | **DF** | **MS** | **F (DFn, DFd)** | **P value** |
| --- | --- | --- | --- | --- | --- |
| Interaction | 9696 | 1 | 9696 | F (1, 12) = 7.915 | P = 0.0157 |
| IRR | 1569 | 1 | 1569 | F (1, 12) = 1.281 | P = 0.2799 |
| 5-ITU | 338.1 | 1 | 338.1 | F (1, 12) = 0.2760 | P = 0.6089 |
| Residual | 14700 | 12 | 1225 |  |  |

5mC 2h protection: CA1

| **ANOVA table** | **SS** | **DF** | **MS** | **F (DFn, DFd)** | **P value** |
| --- | --- | --- | --- | --- | --- |
| Interaction | 5641 | 1 | 5641 | F (1, 12) = 18.58 | P = 0.0010 |
| IRR | 1541 | 1 | 1541 | F (1, 12) = 5.075 | P = 0.0438 |
| 5-ITU | 75.09 | 1 | 75.09 | F (1, 12) = 0.2474 | P = 0.6279 |
| Residual | 3643 | 12 | 303.6 |  |  |

5mC 24h protection: DG

| **ANOVA table** | **SS** | **DF** | **MS** | **F (DFn, DFd)** | **P value** |
| --- | --- | --- | --- | --- | --- |
| Interaction | 46379 | 1 | 46379 | F (1, 12) = 35.70 | P < 0.0001 |
| IRR | 52438 | 1 | 52438 | F (1, 12) = 40.37 | P < 0.0001 |
| 5-ITU | 41347 | 1 | 41347 | F (1, 12) = 31.83 | P = 0.0001 |
| Residual | 15588 | 12 | 1299 |  |  |

5mC 24h protection: CA1

| **ANOVA table** | **SS** | **DF** | **MS** | **F (DFn, DFd)** | **P value** |
| --- | --- | --- | --- | --- | --- |
| Interaction | 5458 | 1 | 5458 | F (1, 12) = 6.058 | P = 0.0300 |
| IRR | 16368 | 1 | 16368 | F (1, 12) = 18.17 | P = 0.0011 |
| 5-ITU | 30358 | 1 | 30358 | F (1, 12) = 33.69 | P < 0.0001 |
| Residual | 10813 | 12 | 901.1 |  |  |

5mC 1mo protection: DG

| **ANOVA table** | **SS** | **DF** | **MS** | **F (DFn, DFd)** | **P value** |
| --- | --- | --- | --- | --- | --- |
| Interaction | 9995 | 1 | 9995 | F (1, 12) = 4.713 | P = 0.0507 |
| IRR | 31414 | 1 | 31414 | F (1, 12) = 14.81 | P = 0.0023 |
| 5-ITU | 25020 | 1 | 25020 | F (1, 12) = 11.80 | P = 0.0049 |
| Residual | 25445 | 12 | 2120 |  |  |

5mC 1mo protection: CA1

| **ANOVA table** | **SS** | **DF** | **MS** | **F (DFn, DFd)** | **P value** |
| --- | --- | --- | --- | --- | --- |
| Interaction | 12291 | 1 | 12291 | F (1, 12) = 10.37 | P = 0.0073 |
| IRR | 18002 | 1 | 18002 | F (1, 12) = 15.19 | P = 0.0021 |
| 5-ITU | 33413 | 1 | 33413 | F (1, 12) = 28.19 | P = 0.0002 |
| Residual | 14221 | 12 | 1185 |  |  |

5mC 1mo mitigation: DG

| **ANOVA table** | **SS** | **DF** | **MS** | **F (DFn, DFd)** | **P value** |
| --- | --- | --- | --- | --- | --- |
| Interaction | 3411 | 1 | 3411 | F (1, 20) = 9.368 | P = 0.0062 |
| IRR | 3541 | 1 | 3541 | F (1, 20) = 9.726 | P = 0.0054 |
| 5-ITU | 22352 | 1 | 22352 | F (1, 20) = 61.39 | P < 0.0001 |
| Residual | 7282 | 20 | 364.1 |  |  |

5mC 1mo mitigation: CA1

| **ANOVA table** | **SS** | **DF** | **MS** | **F (DFn, DFd)** | **P value** |
| --- | --- | --- | --- | --- | --- |
| Interaction | 263.6 | 1 | 263.6 | F (1, 20) = 0.2460 | P = 0.6253 |
| IRR | 7806 | 1 | 7806 | F (1, 20) = 7.283 | P = 0.0138 |
| 5-ITU | 4769 | 1 | 4769 | F (1, 20) = 4.450 | P = 0.0477 |
| Residual | 21434 | 20 | 1072 |  |  |

5hmC 2h protection: DG

| **ANOVA table** | **SS** | **DF** | **MS** | **F (DFn, DFd)** | **P value** |
| --- | --- | --- | --- | --- | --- |
| Interaction | 3.946 | 1 | 3.946 | F (1, 12) = 0.01378 | P = 0.9085 |
| IRR | 556.6 | 1 | 556.6 | F (1, 12) = 1.944 | P = 0.1885 |
| 5-ITU | 2380 | 1 | 2380 | F (1, 12) = 8.312 | P = 0.0138 |
| Residual | 3436 | 12 | 286.3 |  |  |

5hmC 2h protection: CA1

| **ANOVA table** | **SS** | **DF** | **MS** | **F (DFn, DFd)** | **P value** |
| --- | --- | --- | --- | --- | --- |
| Interaction | 75.05 | 1 | 75.05 | F (1, 12) = 0.08754 | P = 0.7724 |
| IRR | 1392 | 1 | 1392 | F (1, 12) = 1.624 | P = 0.2266 |
| 5-ITU | 797.4 | 1 | 797.4 | F (1, 12) = 0.9301 | P = 0.3539 |
| Residual | 10288 | 12 | 857.3 |  |  |

5hmC 24h protection: DG

| **ANOVA table** | **SS** | **DF** | **MS** | **F (DFn, DFd)** | **P value** |
| --- | --- | --- | --- | --- | --- |
| Interaction | 836.6 | 1 | 836.6 | F (1, 12) = 0.9030 | P = 0.3607 |
| IRR | 2346 | 1 | 2346 | F (1, 12) = 2.533 | P = 0.1375 |
| 5-ITU | 2067 | 1 | 2067 | F (1, 12) = 2.231 | P = 0.1611 |
| Residual | 11118 | 12 | 926.5 |  |  |

5hmC 24h protection: CA1

| **ANOVA table** | **SS** | **DF** | **MS** | **F (DFn, DFd)** | **P value** |
| --- | --- | --- | --- | --- | --- |
| Interaction | 211.5 | 1 | 211.5 | F (1, 12) = 0.6105 | P = 0.4498 |
| IRR | 652.5 | 1 | 652.5 | F (1, 12) = 1.883 | P = 0.1951 |
| 5-ITU | 1102 | 1 | 1102 | F (1, 12) = 3.182 | P = 0.0998 |
| Residual | 4158 | 12 | 346.5 |  |  |

5hmC 1mo protection: DG

| **ANOVA table** | **SS** | **DF** | **MS** | **F (DFn, DFd)** | **P value** |
| --- | --- | --- | --- | --- | --- |
| Interaction | 46223 | 1 | 46223 | F (1, 12) = 43.55 | P < 0.0001 |
| IRR | 22395 | 1 | 22395 | F (1, 12) = 21.10 | P = 0.0006 |
| 5-ITU | 56654 | 1 | 56654 | F (1, 12) = 53.38 | P < 0.0001 |
| Residual | 12737 | 12 | 1061 |  |  |

5hmC 1mo protection: CA1

| **ANOVA table** | **SS** | **DF** | **MS** | **F (DFn, DFd)** | **P value** |
| --- | --- | --- | --- | --- | --- |
| Interaction | 3255 | 1 | 3255 | F (1, 12) = 2.285 | P = 0.1565 |
| IRR | 454.6 | 1 | 454.6 | F (1, 12) = 0.3193 | P = 0.5825 |
| 5-ITU | 5412 | 1 | 5412 | F (1, 12) = 3.800 | P = 0.0750 |
| Residual | 17089 | 12 | 1424 |  |  |

5hmC 1mo mitigation: DG

| **ANOVA table** | **SS** | **DF** | **MS** | **F (DFn, DFd)** | **P value** |
| --- | --- | --- | --- | --- | --- |
| Interaction | 6088 | 1 | 6088 | F (1, 12) = 6.855 | P = 0.0225 |
| IRR | 10595 | 1 | 10595 | F (1, 12) = 11.93 | P = 0.0048 |
| 5-ITU | 4131 | 1 | 4131 | F (1, 12) = 4.651 | P = 0.0520 |
| Residual | 10658 | 12 | 888.2 |  |  |

5hmC 1mo mitigation: CA1

| **ANOVA table** | **SS** | **DF** | **MS** | **F (DFn, DFd)** | **P value** |
| --- | --- | --- | --- | --- | --- |
| Interaction | 3917 | 1 | 3917 | F (1, 12) = 9.905 | P = 0.0084 |
| IRR | 2181 | 1 | 2181 | F (1, 12) = 5.515 | P = 0.0368 |
| 5-ITU | 799.6 | 1 | 799.6 | F (1, 12) = 2.022 | P = 0.1805 |
| Residual | 4745 | 12 | 395.4 |  |  |

DNMT3A 1mo protection: DG

| **ANOVA table** | **SS** | **DF** | **MS** | **F (DFn, DFd)** | **P value** |
| --- | --- | --- | --- | --- | --- |
| Interaction | 67039 | 1 | 67039 | F (1, 20) = 73.12 | P < 0.0001 |
| IRR | 79294 | 1 | 79294 | F (1, 20) = 86.49 | P < 0.0001 |
| 5-ITU | 48603 | 1 | 48603 | F (1, 20) = 53.01 | P < 0.0001 |
| Residual | 18336 | 20 | 916.8 |  |  |

DNMT3A 1mo protection: CA1

| **ANOVA table** | **SS** | **DF** | **MS** | **F (DFn, DFd)** | **P value** |
| --- | --- | --- | --- | --- | --- |
| Interaction | 33703 | 1 | 33703 | F (1, 20) = 24.39 | P < 0.0001 |
| IRR | 32843 | 1 | 32843 | F (1, 20) = 23.76 | P < 0.0001 |
| 5-ITU | 29501 | 1 | 29501 | F (1, 20) = 21.35 | P = 0.0002 |
| Residual | 27641 | 20 | 1382 |  |  |

DNMT3A 1mo mitigation: DG

| **ANOVA table** | **SS** | **DF** | **MS** | **F (DFn, DFd)** | **P value** |
| --- | --- | --- | --- | --- | --- |
| Interaction | 40258 | 1 | 40258 | F (1, 20) = 55.48 | P < 0.0001 |
| IRR | 37660 | 1 | 37660 | F (1, 20) = 51.90 | P < 0.0001 |
| 5-ITU | 29303 | 1 | 29303 | F (1, 20) = 40.38 | P < 0.0001 |
| Residual | 14513 | 20 | 725.7 |  |  |

DNMT3A 1mo mitigation: CA1

| **ANOVA table** | **SS** | **DF** | **MS** | **F (DFn, DFd)** | **P value** |
| --- | --- | --- | --- | --- | --- |
| Interaction | 15281 | 1 | 15281 | F (1, 20) = 54.59 | P < 0.0001 |
| IRR | 10845 | 1 | 10845 | F (1, 20) = 38.74 | P < 0.0001 |
| 5-ITU | 12497 | 1 | 12497 | F (1, 20) = 44.65 | P < 0.0001 |
| Residual | 5598 | 20 | 279.9 |  |  |

TET1 2h protection: DG

| **ANOVA table** | **SS** | **DF** | **MS** | **F (DFn, DFd)** | **P value** |
| --- | --- | --- | --- | --- | --- |
| Interaction | 240 | 1 | 240 | F (1, 12) = 3.239 | P = 0.0971 |
| IRR | 1046 | 1 | 1046 | F (1, 12) = 14.12 | P = 0.0027 |
| 5-ITU | 523.8 | 1 | 523.8 | F (1, 12) = 7.069 | P = 0.0208 |
| Residual | 889.2 | 12 | 74.1 |  |  |

TET1 2h protection: CA1

| **ANOVA table** | **SS** | **DF** | **MS** | **F (DFn, DFd)** | **P value** |
| --- | --- | --- | --- | --- | --- |
| Interaction | 5468 | 1 | 5468 | F (1, 12) = 19.63 | P = 0.0008 |
| IRR | 8682 | 1 | 8682 | F (1, 12) = 31.17 | P = 0.0001 |
| 5-ITU | 9439 | 1 | 9439 | F (1, 12) = 33.88 | P < 0.0001 |
| Residual | 3343 | 12 | 278.6 |  |  |

TET1 24h protection: DG

| **ANOVA table** | **SS** | **DF** | **MS** | **F (DFn, DFd)** | **P value** |
| --- | --- | --- | --- | --- | --- |
| Interaction | 332.7 | 1 | 332.7 | F (1, 12) = 0.6143 | P = 0.4484 |
| IRR | 27936 | 1 | 27936 | F (1, 12) = 51.59 | P < 0.0001 |
| 5-ITU | 17988 | 1 | 17988 | F (1, 12) = 33.22 | P < 0.0001 |
| Residual | 6499 | 12 | 541.5 |  |  |

TET1 24h protection: CA1

| **ANOVA table** | **SS** | **DF** | **MS** | **F (DFn, DFd)** | **P value** |
| --- | --- | --- | --- | --- | --- |
| Interaction | 895.8 | 1 | 895.8 | F (1, 12) = 1.240 | P = 0.2873 |
| IRR | 2227 | 1 | 2227 | F (1, 12) = 3.083 | P = 0.1046 |
| 5-ITU | 7343 | 1 | 7343 | F (1, 12) = 10.17 | P = 0.0078 |
| Residual | 8668 | 12 | 722.4 |  |  |

TET1 1mo protection: DG

| **ANOVA table** | **SS** | **DF** | **MS** | **F (DFn, DFd)** | **P value** |
| --- | --- | --- | --- | --- | --- |
| Interaction | 820.2 | 1 | 820.2 | F (1, 12) = 23.26 | P = 0.0004 |
| IRR | 1732 | 1 | 1732 | F (1, 12) = 49.12 | P < 0.0001 |
| 5-ITU | 1189 | 1 | 1189 | F (1, 12) = 33.71 | P < 0.0001 |
| Residual | 423.2 | 12 | 35.27 |  |  |

TET1 1mo protection: CA1

| **ANOVA table** | **SS** | **DF** | **MS** | **F (DFn, DFd)** | **P value** |
| --- | --- | --- | --- | --- | --- |
| Interaction | 744.5 | 1 | 744.5 | F (1, 12) = 14.76 | P = 0.0023 |
| IRR | 3778 | 1 | 3778 | F (1, 12) = 74.90 | P < 0.0001 |
| 5-ITU | 2432 | 1 | 2432 | F (1, 12) = 48.22 | P < 0.0001 |
| Residual | 605.2 | 12 | 50.44 |  |  |

TET1 1mo mitigation: DG

| **ANOVA table** | **SS** | **DF** | **MS** | **F (DFn, DFd)** | **P value** |
| --- | --- | --- | --- | --- | --- |
| Interaction | 22364 | 1 | 22364 | F (1, 12) = 26.97 | P = 0.0002 |
| IRR | 12462 | 1 | 12462 | F (1, 12) = 15.03 | P = 0.0022 |
| 5-ITU | 1947 | 1 | 1947 | F (1, 12) = 2.347 | P = 0.1514 |
| Residual | 9952 | 12 | 829.3 |  |  |

TET1 1mo mitigation: CA1

| **ANOVA table** | **SS** | **DF** | **MS** | **F (DFn, DFd)** | **P value** |
| --- | --- | --- | --- | --- | --- |
| Interaction | 40292 | 1 | 40292 | F (1, 12) = 52.78 | P < 0.0001 |
| IRR | 25860 | 1 | 25860 | F (1, 12) = 33.88 | P < 0.0001 |
| 5-ITU | 13075 | 1 | 13075 | F (1, 12) = 17.13 | P = 0.0014 |
| Residual | 9160 | 12 | 763.3 |  |  |

TET3 2h protection: DG

| **ANOVA table** | **SS** | **DF** | **MS** | **F (DFn, DFd)** | **P value** |
| --- | --- | --- | --- | --- | --- |
| Interaction | 1756 | 1 | 1756 | F (1, 12) = 6.989 | P = 0.0214 |
| IRR | 1332 | 1 | 1332 | F (1, 12) = 5.298 | P = 0.0401 |
| 5-ITU | 61.62 | 1 | 61.62 | F (1, 12) = 0.2452 | P = 0.6294 |
| Residual | 3016 | 12 | 251.3 |  |  |

TET3 2h protection: CA1

| **ANOVA table** | **SS** | **DF** | **MS** | **F (DFn, DFd)** | **P value** |
| --- | --- | --- | --- | --- | --- |
| Interaction | 9995 | 1 | 9995 | F (1, 16) = 26.94 | P < 0.0001 |
| IRR | 12326 | 1 | 12326 | F (1, 16) = 33.22 | P < 0.0001 |
| 5-ITU | 393.4 | 1 | 393.4 | F (1, 16) = 1.060 | P = 0.3185 |
| Residual | 5937 | 16 | 371.1 |  |  |

TET3 24h protection: DG

| **ANOVA table** | **SS** | **DF** | **MS** | **F (DFn, DFd)** | **P value** |
| --- | --- | --- | --- | --- | --- |
| Interaction | 13.84 | 1 | 13.84 | F (1, 12) = 0.6862 | P = 0.4236 |
| IRR | 14.9 | 1 | 14.9 | F (1, 12) = 0.7389 | P = 0.4069 |
| 5-ITU | 257.9 | 1 | 257.9 | F (1, 12) = 12.79 | P = 0.0038 |
| Residual | 242 | 12 | 20.17 |  |  |

TET3 24h protection: CA1

| **ANOVA table** | **SS** | **DF** | **MS** | **F (DFn, DFd)** | **P value** |
| --- | --- | --- | --- | --- | --- |
| Interaction | 290.7 | 1 | 290.7 | F (1, 12) = 1.305 | P = 0.2756 |
| IRR | 771.2 | 1 | 771.2 | F (1, 12) = 3.461 | P = 0.0875 |
| 5-ITU | 454.1 | 1 | 454.1 | F (1, 12) = 2.038 | P = 0.1789 |
| Residual | 2674 | 12 | 222.8 |  |  |

TET3 1mo protection: DG

| **ANOVA table** | **SS** | **DF** | **MS** | **F (DFn, DFd)** | **P value** |
| --- | --- | --- | --- | --- | --- |
| Interaction | 788 | 1 | 788 | F (1, 12) = 26.60 | P = 0.0002 |
| IRR | 573.1 | 1 | 573.1 | F (1, 12) = 19.34 | P = 0.0009 |
| 5-ITU | 814.8 | 1 | 814.8 | F (1, 12) = 27.50 | P = 0.0002 |
| Residual | 355.5 | 12 | 29.63 |  |  |

TET3 1mo protection: CA1

| **ANOVA table** | **SS** | **DF** | **MS** | **F (DFn, DFd)** | **P value** |
| --- | --- | --- | --- | --- | --- |
| Interaction | 1560 | 1 | 1560 | F (1, 12) = 18.47 | P = 0.0010 |
| IRR | 4199 | 1 | 4199 | F (1, 12) = 49.75 | P < 0.0001 |
| 5-ITU | 1352 | 1 | 1352 | F (1, 12) = 16.02 | P = 0.0018 |
| Residual | 1013 | 12 | 84.42 |  |  |

TET3 1mo mitigation: DG

| **ANOVA table** | **SS** | **DF** | **MS** | **F (DFn, DFd)** | **P value** |
| --- | --- | --- | --- | --- | --- |
| Interaction | 5553 | 1 | 5553 | F (1, 12) = 13.87 | P = 0.0029 |
| IRR | 5504 | 1 | 5504 | F (1, 12) = 13.75 | P = 0.0030 |
| 5-ITU | 3681 | 1 | 3681 | F (1, 12) = 9.195 | P = 0.0104 |
| Residual | 4804 | 12 | 400.3 |  |  |

TET3 1mo mitigation: CA1

| **ANOVA table** | **SS** | **DF** | **MS** | **F (DFn, DFd)** | **P value** |
| --- | --- | --- | --- | --- | --- |
| Interaction | 15360 | 1 | 15360 | F (1, 12) = 188.1 | P < 0.0001 |
| IRR | 13559 | 1 | 13559 | F (1, 12) = 166.0 | P < 0.0001 |
| 5-ITU | 5994 | 1 | 5994 | F (1, 12) = 73.39 | P < 0.0001 |
| Residual | 980.1 | 12 | 81.67 |  |  |

**Molecular markers: F(Dfn, Dfd), P-values**

­­­­­­­­­­­­­­­

**ADK 2hr Protection:**

F (3, 11) = 5.660

P = 0.0136

**ADK 1mo Protection:**

F (3, 11) = 73.43

P < 0.0001

**ADK 1mo Mitigation:**

F (3, 11) = 14.70

P = 0.0004

**5mC 2hr Protection CA1:**

F (3, 11) = 7.968

P = 0.0035

**5mC 2hr Protection DG:**

F (3, 11) = 3.157

P = 0.0644

**5mC 24hr Protection CA1**:

F (3, 11) = 19.30

P < 0.0001

**5mC 24hr Protection DG:**

F (3, 11) = 35.97

P < 0.0001

**5mC 1mo Protection CA1:**

F (3, 11) = 17.92

P < 0.0001

**5mC 1mo Protection DG:**

F (3, 11) = 10.44

P = 0.0012

**5mC 1mo Mitigation CA1:**

F (3, 11) = 3.993

P = 0.0348

**5mC 1mo Mitigation DG:**

F (3, 11) = 26.83

P < 0.0001

**5hmC 2h Protection CA1:**

F (3, 11) = 1.045

P = 0.4147

**5hmC 2h Protection DG:**

F (3, 11) = 3.311

P = 0.0655

**5hmC 24h Protection CA1:**

F (3, 11) = 1.758

P = 0.2184

**5hmC 24h Protection DG:**

F (3, 11) = 1.650

P = 0.2398

**5hmC 1mo Protection CA1:**

F (3, 11) = 2.135

P = 0.1491

**5hmC 1mo Protection DG:**

F (3, 11) = 39.34

P < 0.0001

**5hmC 1mo Mitigation CA1:**

F (3, 11) = 5.814

P = 0.0108

**5hmC 1mo Mitigation DG:**

F (3, 11) = 7.811

P = 0.0037

**DNMT3A 1mo Protection CA1:**

F (3, 11) = 23.17

P < 0.0001

**DNMT3A 1mo Protection DG:**

F (3, 11) = 70.87

P < 0.0001

**DNMT3A 1mo Mitigation CA1:**

F (3, 11) = 45.99

P < 0.0001

**DNMT3A 1mo Mitigation DG:**

F (3, 11) = 49.25

P < 0.0001

**TET1 2hr Protection CA1:**

F (3, 11) = 28.23

P < 0.0001

**TET1 2hr Protection DG:**

F (3, 11) = 8.143

P = 0.0016

**TET1 24hr Protection CA1:**

F (3, 11) = 4.829

P = 0.0198

**TET1 24hr Protection DG:**

F (3, 11) = 28.47

P < 0.0001

**TET1 1mo Protection CA1:**

F (3, 11) = 45.96

P < 0.0001

**TET1 1mo Protection DG:**

F (3, 11) = 35.37

P < 0.0001

**TET1 1mo Mitigation CA1:**

F (3, 11) = 34.60

P < 0.0001

**TET1 1mo Mitigation DG:**

F (3, 11) = 14.78

P = 00.0002

­­­­­­­­­­­­­­­­­­

**TET3 2hr Protection CA1:**

F (3, 11) = 20.40

P < 0.0001

**TET3 2hr Protection DG:**

F (3, 11) = 4.117

P = 0.0306

**TET3 24hr Protection CA1:**

F (3, 11) = 2.125

P = 0.1605

**TET3 24hr Protection DG:**

F (3, 11) = 4.109

P = 0.0386

**TET3 1mo Protection CA1:**

F (3, 11) = 28.08

P < 0.0001

**TET3 1mo Protection DG:**

F (3, 11) = 24.48

P < 0.0001

**TET3 1mo Mitigation CA1:**

F (3, 11) = 142.5

P < 0.0001

**TET3 1mo Mitigation DG:**

F (3, 11) = 12.27

P = 0.0006

­­­­­­­­­­­­­­­­­­­
